# Lateral Habenula Mediates Defensive Responses Only When Threat and Safety Memories Are in Conflict

**DOI:** 10.1523/ENEURO.0482-20.2021

**Published:** 2021-04-19

**Authors:** Geronimo Velazquez-Hernandez, Francisco Sotres-Bayon

**Affiliations:** Cell Physiology Institute–Neuroscience, National Autonomous University of Mexico, Mexico City 04510, Mexico

**Keywords:** conflict, decision-making, emotion, fear, habenula, motivation

## Abstract

Survival depends on the ability to adaptively react or execute actions based on previous aversive salient experiences. Although lateral habenula (LHb) activity has been broadly implicated in the regulation of aversively motivated responses, it is not clear under which conditions this brain structure is necessary to regulate defensive responses to a threat. To address this issue, we combined pharmacological inactivations with behavioral tasks that involve aversive and appetitive events and evaluated defensive responses in rats. We found that LHb pharmacological inactivation did not affect cued threat conditioning (fear) and extinction (safety) learning and memory, anxiety-like or reward-seeking behaviors. Surprisingly, we found that LHb inactivation abolished reactive defensive responses (tone-elicited freezing) only when threat (conditioning) and safety memories (extinction and latent inhibition) compete during retrieval. Consistently, we found that LHb inactivation impaired active defensive responses [platform-mediated avoidance (PMA)], thereby biasing choice behavior (between avoiding a threat or approaching food) toward reward-seeking responses. Together, our findings suggest that LHb activity mediates defensive responses only when guided by competing threat and safety memories, consequently revealing a previously uncharacterized role for LHb in experience-dependent emotional conflict.

## Visual Abstract

**Figure:**
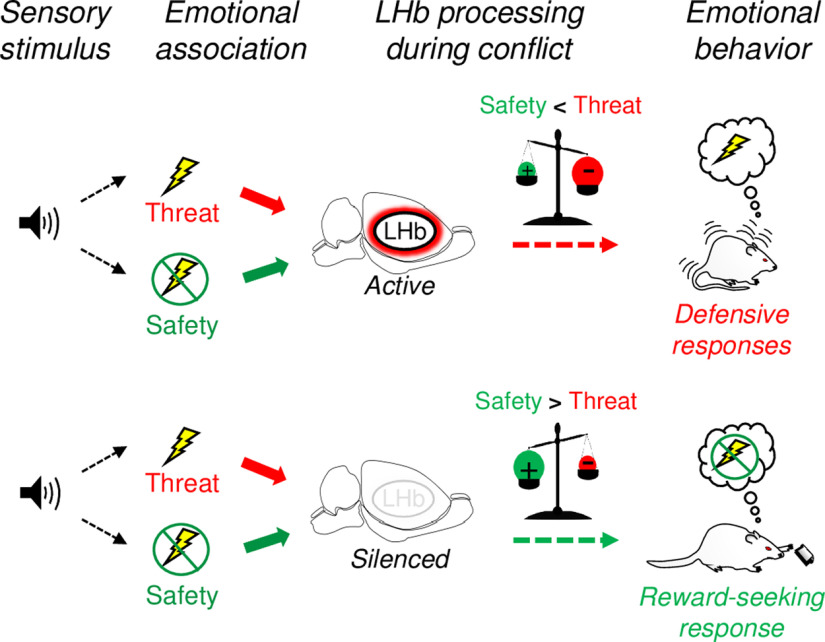

## Significance Statement

Using emotional memories with opposing valence (negative or positive) to select the appropriate behavior is most valuable for survival when individuals are challenged to retrieve them simultaneously, during conflict. Although the lateral habenula (LHb) processes information about negative and positive events and is involved in guiding emotional behaviors, its contribution during conflict has not been characterized. By combining pharmacological inactivations with behavioral paradigms in rats, including tasks involving retrieval of competing threat and safety memories, our study reveals a previously unknown role for the LHb in driving defensive responses during experience-dependent emotional conflict. Identifying brain regions required for conflict-mediated behavioral responses may be key for understanding how the brain balances information about competing salient experiences to regulate emotional behaviors.

## Introduction

In nature, survival in a dynamic environment requires animals to adaptively react or execute actions based on previous salient experiences (Elliot, 2008; Rangel et al., 2008; Namburi et al., 2016; Bravo-Rivera and Sotres-Bayon, 2020). In the laboratory, the regulation of reactive and active behaviors guided by stimuli that signal threat or safety and reward have been studied by using animal model combinations of aversive classical (Pavlovian) and appetitive instrumental conditioning (LeDoux, 2000; Cardinal et al., 2002). Auditory threat (fear) conditioning, in combination with lever pressing to obtain food, has been used to reliably evaluate defensive responses elicited after learning and memory of threat associations (tones paired with shocks) and its extinction (tones paired with omission of expected shocks; Quirk et al., 2000; Sotres-Bayon et al., 2012). After extinction, the conditioned memory of threat and extinction memory of safety, coexist in the brain and compete for control of behavior (Quirk, 2007; Sotres-Bayon and Quirk, 2010; Lacagnina et al., 2019). Other experimental conditions that can be used to study the conflict between threat and safety memories is conditioned latent inhibition, which involves threat association of a tone that was previously associated with safety (Lingawi et al., 2017), as well as more recently developed tasks that involve the use of conflict choice behavior (Bravo-Rivera and Sotres-Bayon, 2020), such as platform-mediated avoidance (PMA; Diehl et al., 2019). The use of these behavioral paradigms can help us characterize how the brain retrieves emotional memories to adaptively choose a defensive behavioral strategy.

The lateral habenula (LHb) has recently attracted much attention for its involvement in the regulation of a variety of behavioral responses triggered by aversive events (Hikosaka, 2010; Lammel et al., 2014; Proulx et al., 2014; Baker et al., 2016), including threat avoidance (Stamatakis and Stuber, 2012; Amo et al., 2014; Vincenz et al., 2017; Trusel et al., 2019; Stephenson-Jones et al., 2020), freezing (Martínez-Canabal et al., 2019), and choice behavior bias (Stopper and Floresco, 2014; Baker et al., 2017). The broad involvement of LHb in processing of aversive events is partly explained by its widely documented increase of activity in response to a variety of adverse stimuli, including those that were initially neutral but that have been conditioned to predict threatening events (Matsumoto and Hikosaka, 2009; Trusel et al., 2019). However, it is not clear whether LHb is necessary to control threat-related behavioral responses ubiquitously. LHb lesions or pharmacological inactivations facilitated (Song et al., 2017), impaired (Thornton and Bradbury, 1989), or had no effect (Vale-Martínez et al., 1997; Shumake et al., 2010; Ilango et al., 2013) on threat avoidance. In addition, LHb contribution to another commonly evaluated defensive response, such as freezing, have been scarcely explored (Heldt and Ressler, 2006). Importantly, although LHb is predominantly involved with learning from negative events (Matsumoto and Hikosaka, 2009; Tian and Uchida, 2015), it has also been shown to signal positive value information (Stamatakis and Stuber, 2012). LHb neurons decrease their activity to positive stimuli, including those stimuli that have been conditioned to predict reward (Matsumoto and Hikosaka, 2009) and events that may signal safety through omission of threat (Li et al., 2019). Thus, given that LHb appears to simultaneously processes information about negative and positive events to guide motivated behaviors, it is possible that this brain region is critically involved under conditions that challenge individuals to convey information about opposing acquired values to adaptably control defensive responses.

To fully characterize LHb requirement in the control of defensive responses, we combined pharmacological inactivations with behavioral paradigms that involve threat stimuli, appetitive events and their competition. Surprisingly, we found that LHb activity is critical to promote reactive (freezing to a tone) and active (avoidance to a tone) defensive response only when threat (conditioning) and safety (extinction and latent inhibition) memories are in conflict.

## Materials and Methods

### Subjects

A total of 144 adult male Wistar rats (Cell Physiology Institute breeding colony) and weighing 260–280 g were housed in polyethylene cages and maintained on a standard 12/12 h light/dark cycle. Rats were housed individually, handled daily to diminish stress responses and received water *ad libitum* during all experiments. Rats were kept on a restricted diet (12 g/d) of standard laboratory rat chow to facilitate pressing a lever for sucrose pellets (Bio-Serv). All manipulations and behavioral procedures were performed during the light phase between 7 A.M. and 7 P.M. All procedures were approved by the Institutional Animal Care and Use Committee of the National Autonomous University of Mexico (UNAM) in compliance with the National Ministry of Health guidelines for the care of laboratory animals.

### Surgery

Rats were anesthetized with isoflurane inhalant gas and implanted with 23-gauge stainless steel guide cannulas (AM Systems) targeting the lateral region of the habenula (−3.6 mm, AP, ±0.8 mm, −4.2 mm DV with respect to bregma from skull). Cannulas were fixed with dental acrylic cement and anchored with two surgical screws placed on the skull. Stainless steel obturators were inserted into the guide cannulas to prevent clogging until infusions were made. The tips of the cannulas were aimed 0.8 mm above the target structure. After surgery, animals received food and water *ad libitum* for 7 d to allow full recovery before experiments.

### Behavior

Rats were trained to press a lever for sucrose pellets (Bio-Serv) on a variable interval (VI) reinforcement schedule [VI-60 for conditioning tasks as in Sierra-Mercado et al. (2011) and VI-30 for the avoidance task as in Bravo-Rivera et al. (2014)]. All rats received six lever-press training sessions until they reached a minimum rate of 12 presses per minute in their final session. Pressing to obtain food maintains a constant level of activity against which freezing, and avoidance can be reliably measured.

### Threat conditioning and extinction

Threat conditioning and extinction tasks were performed as previously described (Martínez-Canabal et al., 2019). Briefly, auditory threat conditioning and extinction was performed in standard operant chambers (Coulbourn Instruments) located inside sound-attenuating boxes (Med Associates) in an isolated testing room. The floor of the operant chambers consisted of stainless-steel bars that could deliver electric foot-shocks. Between experiments shock grids, floor trays and walls were cleaned with soap and water. On day 1, rats were subjected to threat conditioning consisting of five tone presentations (30 s, 4 kHz, 75 dB) that co-terminated each one with a foot-shock (0.5 s, 0.6 mA). On day 2, after conditioning, rats received extinction training consisting of fifteen presentations of unreinforced tones. On day 3, rats were tested for memory retrieval with two tone-alone presentations. In all sessions, the interval between tones was variable with an averaged 2 min.

### Conditioned latent inhibition

On day 1, rats received latent inhibition training consisting of fifteen tone-alone presentations (30 s, 4 kHz, 75 dB). On day 2, rats were subjected to threat conditioning, consisting of five tones (30 s, 4 Hz, 75 dB) that co-terminated with a foot-shock (0.5 s, 0.6 mA). On day 3, rats were tested for memory retrieval with two tone-alone presentations. In all sessions, the interval between tones was variable with an average of 2 min.

### PMA task

PMA task was performed as previously described (Bravo-Rivera et al., 2014) using standard operant chambers (Coulbourn Instruments) located inside sound-attenuating boxes (Med Associates) in an isolated testing room. The floor of the operant chambers consisted of stainless-steel bars that could deliver electric foot-shocks. Between experiments shock grids, floor trays and walls were cleaned with soap and water. Rats were conditioned to a tone (30 s, 4 kHz, 75 dB) co-terminating with a shock delivered through a grid floor (2 s, 0.4 mA). Rats received nine tone–shock pairings each day for 10 d, with a VI between tones averaging 3 min. A safety square platform (14.0 cm per side) was located in the opposite corner of the lever to allow rats to avoid the shocks. The availability of food on the side opposite of the platform motivated rats to leave the platform during the inter-trial interval, facilitating trial-by-trial assessment of avoidance. On day 11, rats received two tones to test for memory retrieval.

### Open field task

Rat locomotor activity in the open field (90 × 90 cm) was assessed by calculating distance traveled in the periphery and center (30 × 30 cm within) of the arena during 10 min. The overall distance traveled and number of entries to the center of the arena were used to assess locomotion and anxiety-like behavior, respectively.

### Lever pressing test

Rats were trained to press a lever to receive food pellets on a VI-60 schedule (as described above). Spontaneous presses per minute were calculated to assess reward-seeking behavior during 5 min. We analyzed presses per minute before conditioning (during pretone baseline) taken from rats in the first experiment.

### Drug infusion

We used GABA-A and GABA-B receptors agonists, muscimol and baclofen (M&B; Sigma-Aldrich) respectively, to enhance neuronal inhibition, thereby temporarily and reversibly inactivating the target structure. The inactivating drug cocktail was prepared on the day of the infusion using a filtered, physiological saline solution (SAL) as vehicle and infused 10 min before behavioral testing. M&B (50 ng of each drug/0.2 μl per side) or SAL was infused at a rate of 0.2 μl delivered over 45 s into the target structure. Cannulas were connected via polyethylene tubing to 10-μl syringes (Hamilton) driven by a programmable microinfusion pump (KD Scientific). After infusions, injectors were left in place for 2 min to allow the drug to diffuse. Dosages and volumes of GABAergic agonists were based on a previous inactivation study targeting the same brain structure which limited spread to LHb without affecting neighboring areas (Stopper and Floresco, 2014), and a pilot experiment in our laboratory (combining habenula infusions and c-Fos expression) showing that local inactivation of the habenula silenced LHb recruited activity.

### Histology

After all behavioral experiments, rats were deeply anesthetized with pentobarbital sodium (150 mg/kg, i.p.) to transcardially perfused with 0.9% SAL. Brains were then harvested and stored in a 10% formalin solution (Sigma-Aldrich) for at least 2 d. Then we switched the brains to a 30% sucrose solution for cryoprotection. Brains were cut in 45-μm-thick coronal sections using a cryostat (Leica CM1520 Cryostat, RRID:SCR_017543) and stained for Nissl bodies with cresyl violet and examined under a bright field microscope (Nikon, H550S) to verify cannula tip placements. Only animals with localized injector tips within LHb were included in statistical analysis; except for one experiment (LHb inactivation after extinction), in which analysis of data included animals where injector tips were localized within or outside the LHb (hit M&B and miss M&B groups, respectively).

### Data collection and analysis

All behavioral responses were recorded with digital video cameras (Logitech) and automatically analyzed with a commercial software (ANY-maze, RRID:SCR_014289). Two measures of defensive responses were assessed throughout all experiments: (1) % of time spent freezing and (2) suppression ratio. Freezing was defined as the absence of all movement, except respiration. The amount of time freezing to the tone was expressed as the percentage of the tone duration. We also measured time spent freezing before tone presentations (5 min), during context exposure (pretone freezing). There were little contextual-elicited defensive responses across all experiments (SAL and M&B groups), as evidenced by low levels of pretone freezing (all group averages < 12.03%). In all experiments pretone freezing percentages were not significantly different between experimental and control groups (all *p* values > 0.05). Matching of animal groups (experimental and controls) for similar levels of freezing responses before local drug infusions were done in all experiments (*p* = 0.95). A suppression ratio comparing pretone press rates with tone press rates was calculated as follows: (pretone – tone)/(pretone + tone). A value of 0 represents no suppression (low defensive response levels), whereas a value of +1 represents complete suppression of lever pressing (high defensive response levels). Avoidance responses were assessed by calculating the percentage of rats that avoided the footshock by moving on to the platform during the last two seconds of tone presentations and time spent on platform before and after tone presentation. Groups were compared by using, when appropriate, unpaired Student’s two-tailed *t* tests, one-way or repeated-measures ANOVA followed by *post hoc* Fisher’s least significant difference test (STATISTICA; StatSoft; RRID:SCR_014213 and Prism; GraphPad Prism RRID:SCR_002798). Alpha was set at *p *=* *0.05.

## Results

Although LHb activity has been broadly implicated in the regulation of aversively motivated behaviors (Thornton and Bradbury, 1989; Matsumoto and Hikosaka, 2009; Stamatakis and Stuber, 2012; Amo et al., 2014; Lecca et al., 2017; Song et al., 2017; Vincenz et al., 2017; Martínez-Canabal et al., 2019; Trusel et al., 2019), it is not always necessary for regulating defensive responses to a threat (Vale-Martínez et al., 1997; Heldt and Ressler, 2006; Shumake et al., 2010; Ilango et al., 2013; Song et al., 2017). To systematically evaluate the necessity of LHb under different aversive conditions, we combined pharmacological inactivations with behavioral tasks that involve aversive and appetitive motivations and evaluated defensive behavioral responses in rats.

### LHb inactivation does not affect threat or extinction learning and memory

**Figure 1.**
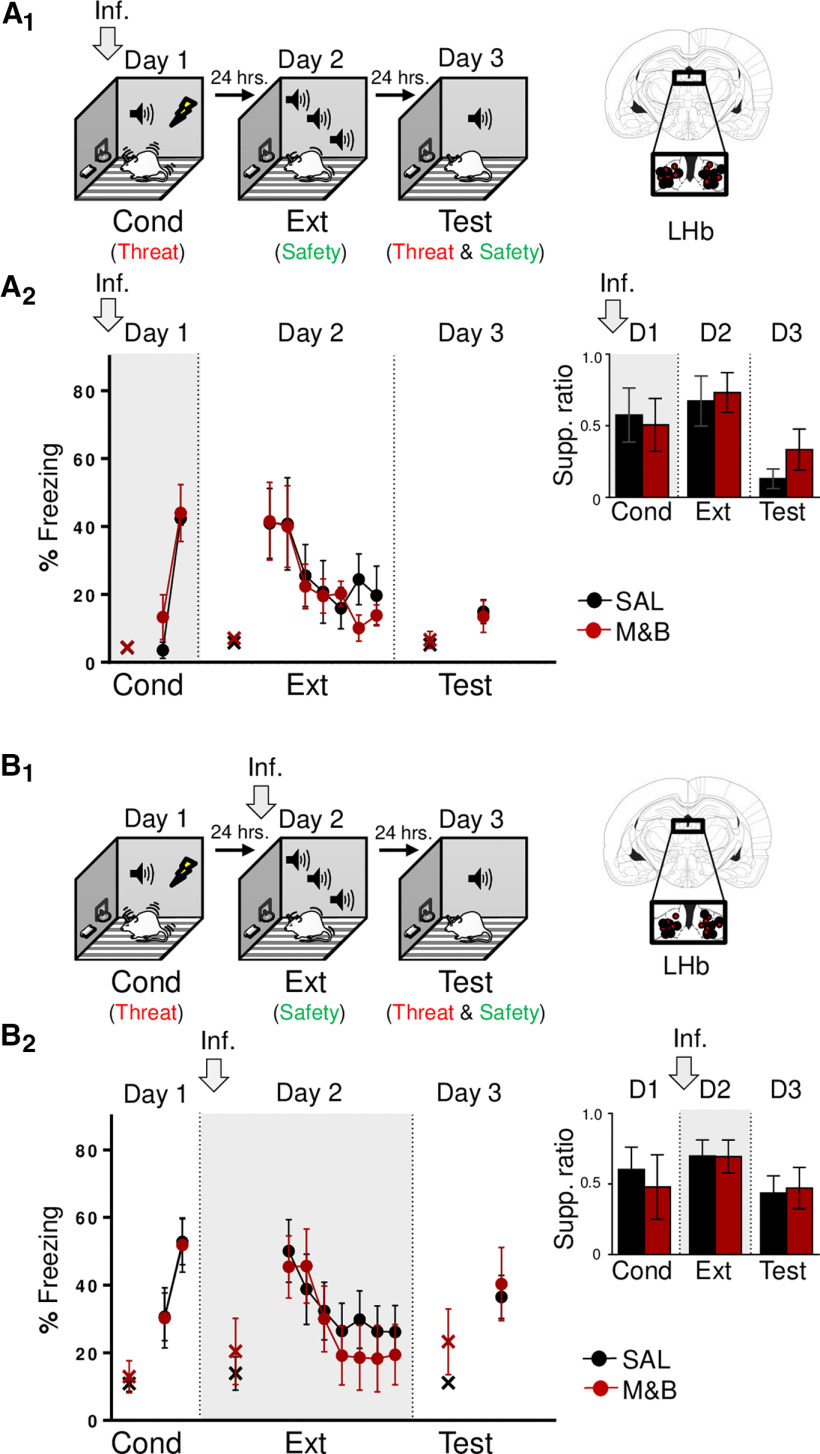
LHb inactivation before threat and extinction training does not affect learning and memory. ***A_1_***, ***B_1_***, left, Schematic representation of rats training. Rats were infused with SAL or M&B into LHb (gray arrow) before being trained to associate a tone with foot-shocks (day 1; Cond, conditioning). This training represents threat learning (red). One day later, rats were trained to associate the same tone with the absence of foot-shocks (day 2; Ext, extinction), which represents safety learning (green). Finally, the next day, rats were presented with tones only (day 3, test), which involves the retrieval competition of coexisting threat (red) and safety (green) memories to control defensive responses. Right, Coronal drawings of rat brain atlas showing placements of the injector tips in LHb. ***A_2_***, LHb inactivation before threat conditioning (day 1; gray arrow and shaded area in graph), did not affect threat learning (day 1) or its memory formation (day 2), as indicated similar levels of tone-elicited freezing and lever-press suppression ratio [inset; late conditioning (last two-tone trial block), early extinction (first two-tone trial block), and test] between groups (SAL, *n* = 5; M&B, *n* = 7). ***B_2_***, Infusion of M&B into LHb before extinction training (day 2; gray arrow and shaded area in graph), did not affect, expression of threat memory (day 2), safety learning (day 2) or its memory formation (day 3), as indicated by similar levels of percent freezing and lever-press suppression ratio [inset, late conditioning (last two-tone trial block), early extinction (first two-tone trial block), and test] between groups (SAL, *n* = 11; M&B, *n* = 9). Data are shown as ±SEM in blocks of two trials. LHb, lateral habenula. Inf, infusion. Supp. ratio, suppression ratio; x, baseline (pretone) freezing levels.

To evaluate the requirement of LHb on threat learning and memory, we pharmacologically inactivated the LHb before auditory threat conditioning on day 1 (Fig. 1*A1*). We found that inactivation of LHb before threat conditioning did not affect threat acquisition or its retrieval (Fig. 1*A2*), as indicated by similar levels of defensive responses across groups (day 1, freezing: *F*_(1,10)_ = 0.71, *p *=* *0.41; lever-press suppression ratio: SAL: *t*_(10)_ = −0.25, *p *=* *0.80) and the first two-tone trial block of extinction training (day 2, freezing: *t*_(10)_ = 0.03, *p *=* *0.96; lever-press suppression ratio: *t*_(10)_ = 0.26, *p *=* *0.79), respectively. Also, LHb inactivation before threat conditioning did not affect extinction acquisition 1 d later or its retrieval 2 d later, as indicated by similar levels of defensive responses across groups in extinction training (day 2, freezing: *F*_(6,60)_ = 0.61, *p *=* *0.71; lever-press suppression ratio: *F*_(6,60)_ = 0.52, *p *=* *0.78) and in the memory test (day 3, freezing: *t*_(10)_ = −0.22, *p *=* *0.82; lever-press suppression ratio: *t*_(10)_ = 1.12, *p *=* *0.28). Thus, consistent with previous reports (Heldt and Ressler, 2006; Song et al., 2017), our findings suggest that LHb is not necessary for threat learning and memory.

We next investigated the necessity of LHb on threat memory retrieval as well as on extinction learning and memory by pharmacologically inactivating the LHb before auditory threat extinction training (Fig. 1*B1*). Rats were infused with either SAL or M&B into the LHb on day 2, before threat extinction training. One day later (day 3), rats were tested for retrieval of emotional memory to the tone. We found that inactivation of LHb before threat extinction did not affect retrieval of threat memory or extinction acquisition (Fig. 1*B2*), as indicated by similar levels of defensive responses across groups in the first block of two-tone trials of extinction training (day 2, freezing: *t*_(18)_ = −0.35, *p *=* *0.72; lever-press suppression ratio: *t*_(18)_ = −0.02, *p *=* *0.97) and overall during extinction training (day 2, freezing: *F*_(6,108)_ = 0.92, *p *=* *0.47; lever-press suppression ratio: *F*_(6,108)_ = 1.90, *p *=* *0.08), respectively. Additionally, consistent with a previous report (Song et al., 2017), we found that LHb inactivation before extinction training, did not affect retrieval of extinction-mediated safety memory 1 d later, as indicated by similar levels of defensive responses across groups during memory test (day 3, freezing: *t*_(18)_ = 0.31, *p *=* *0.75; lever-press suppression ratio: *t*_(18)_ = 0.18, *p *=* *0.85). Thus, these results suggest that LHb is not necessary for threat memory retrieval as well as not necessary for extinction learning and memory. Together our findings indicate that LHb is not necessary for threat and extinction learning and memory.

### LHb inactivation impairs defensive responses during extinction-dependent retrieval

**Figure 2.**
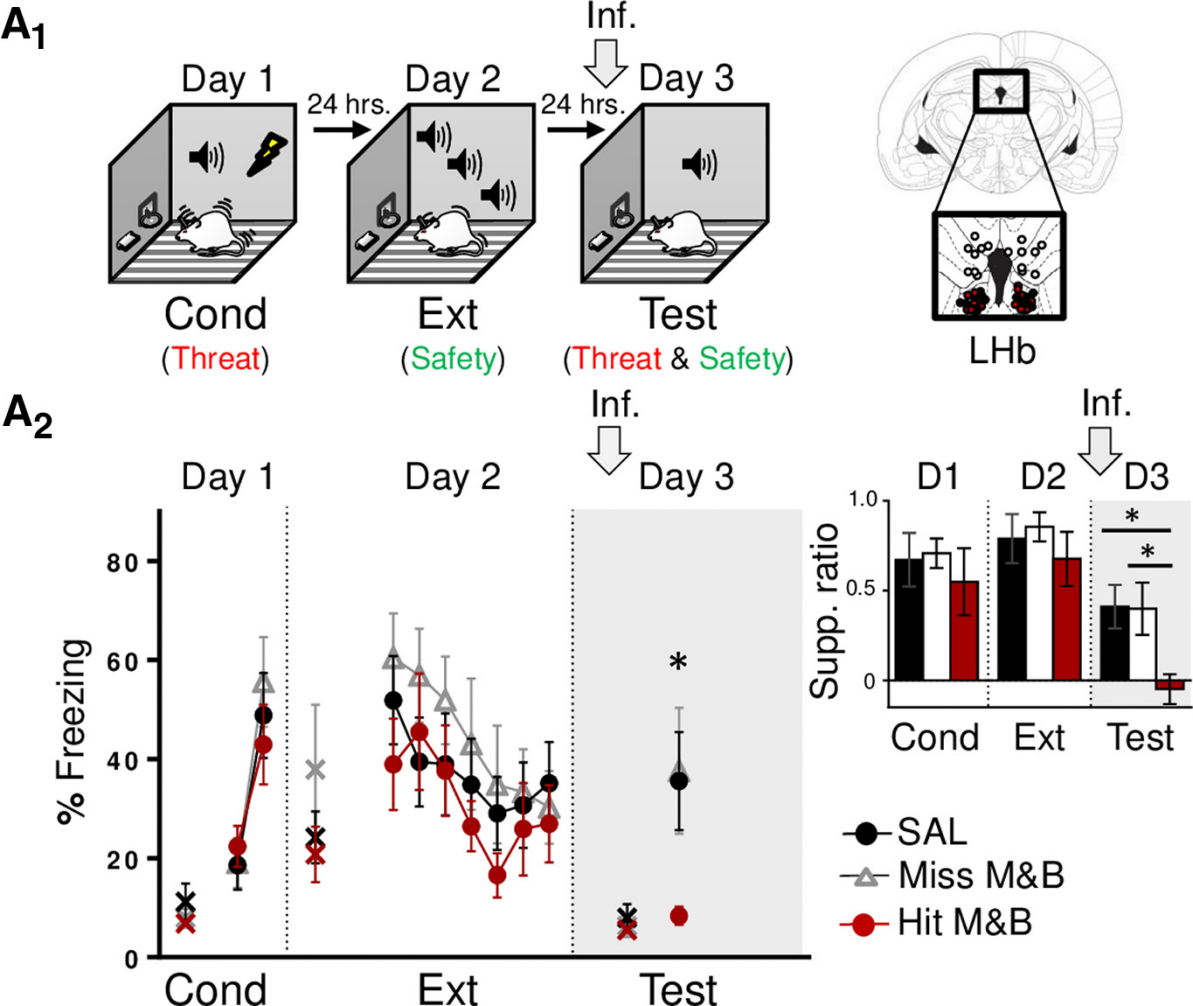
LHb inactivation before memory test, after extinction, abolishes defensive responses. ***A_1_***, left, Schematic representation of rats training. Rats were trained to associate tones with foot-shocks (day 1; Cond, conditioning), which represents threat learning (red). One day later, rats were trained to associate the same tone with the absence of foot-shocks (day 2; Ext, extinction), which represents safety learning (green). Finally, the next day, rats were infused with SAL or M&B into LHb (gray arrow) before being presented with tones only (day 3, test), which involves the retrieval competition of coexisting threat (red) and safety (green) memories to control defensive responses. Right, Coronal drawings of rat brain atlas showing placements of the injector tips in LHb. ***A_2_***, LHb inactivation before retrieval test (day 3), after extinction (gray arrow and shaded area in graph), decreased defensive responses, as indicated by lower levels of tone-elicited percent freezing and lever-press suppression ratio [inset; late conditioning (last two-tone trial block), early extinction (first two-tone trial block), and test] in the experimental group as compared with the control groups (SAL, *n* = 7; miss M&B, *n* = 8; hit M&B, *n* = 9). Data are shown as ± SEM in blocks of two trials. LHb, lateral habenula. Inf, infusion. Supp. ratio, suppression ratio; x, baseline (pretone) freezing levels; *p < 0.05.

To evaluate the necessity of LHb in retrieval events after extinction learning, involving the competition of coexisting conditioned-mediated threat memory and extinction-mediated safety memory, we pharmacologically inactivated the LHb before retrieval memory test on day 3 (Fig. 2*A1*). Surprisingly, we found that LHb inactivation before memory test, after extinction, abolished defensive responses (freezing and suppressed pressing) to preconditioning levels of the experimental group as compared with control group during memory test (day 3, freezing: SAL: 35.59%: M&B: 8.39%; *t*_(14)_ = −3.06, *p *=* *0.008; lever-press suppression ratio: SAL: 0.41; M&B: −0.04; *t*_(14)_ = −3.22, *p *=* *0.006). Based on a previous LHb inactivation study (Stopper and Floresco, 2014) and to further examine the neuroanatomical specificity of the inactivation effect, after histology, data were separated into three groups: one SAL-infused group and two M&B-infused groups. One group represents, a neuroanatomical control, in which placements missed (miss M&B) the targeted brain region (placements included dorsal hippocampus and third ventricle) and another group represents rats where placements directly hit (hit M&B) only in the LHb (day 3, freezing: SAL: 35.59%, miss M&B: 37.64%, hit M&B: 8.39%; *F*_(2,21)_ = 3.54, *p *=* *0.04; *post hoc* comparisons: SAL and hit M&B: *p *=* *0.04, miss M&B and hit M&B: *p *=* *0.02, and SAL and miss M&B: *p *=* *0.87; lever-press suppression ratio: SAL: 0.41, miss M&B: 0.39, hit M&B: −0.04; *F*_(2,21)_ = 5.28, *p *=* *0.01; *post hoc* comparisons: SAL and hit M&B: *p *=* *0.01, miss M&B and hit M&B: *p *=* *0.01, and SAL and miss M&B: *p *=* *0.94; Fig. 2*A2*). Results after comparison between experimental (hit M&B) and control groups (SAL and miss M&B) reveal that the effects of inactivation on memory retrieval after extinction were circumscribed to the LHb, but not nearby regions. That LHb inactivation before the retrieval test decreased defensive responses could be interpreted as an impairment in the expression of defensive responses. However, this is unlikely given that LHb inactivation did not impair defensive response expression when performed before conditioning or before extinction training (see day 1 in Fig. 1*A2* and day 2 in Fig. 1*B2*). Another possible interpretation to this result is that LHb inactivation before testing impaired threat memory retrieval. However, this is also unlikely given that LHb inactivation did not impair threat memory retrieval when performed before extinction training (see first two-tone trial block in day 2 in Fig. 1*B2*). Yet another possibility is that LHb inactivation before the retrieval test facilitated extinction memory thereby biasing the expression of the extinction memory over threat memory. This last interpretation suggests that LHb may be necessary to regulate competing threat and safety memories by promoting defensive responses. But before testing this idea further, we decided to test whether the observed LHb inactivation effect was dependent or not on extinction triggered by the tone (rather than the mere passing of time or context exposure) and also evaluate whether LHb inactivation causes unspecific effects on basic locomotor activity or independent motivated behaviors.

**Figure 3.**
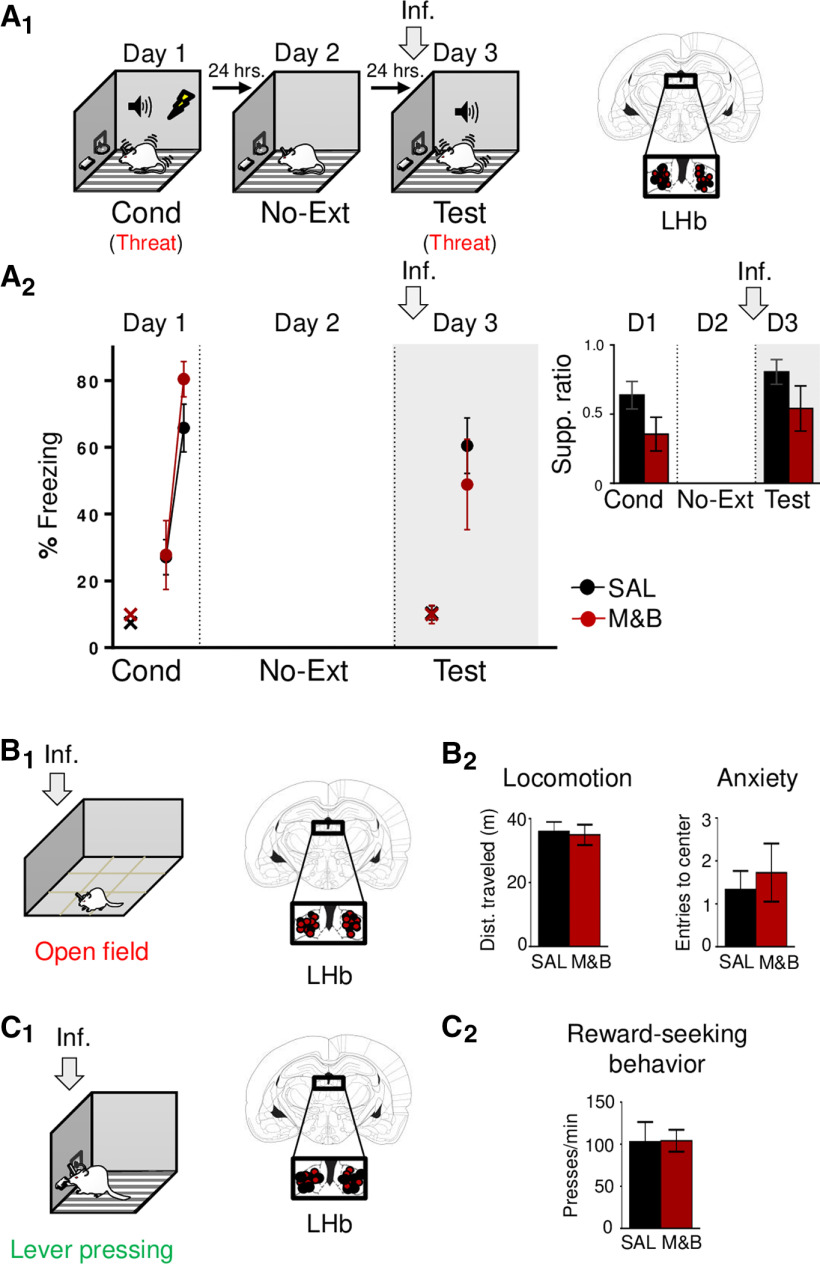
LHb inactivation effect is extinction-dependent and not attributable to impairments in locomotion or other emotional behaviors. ***A_1_***, left, Schematic representation of rats training. Rats were trained to associate a tone with foot-shocks (day 1; Cond, conditioning), which represents threat learning (red). One day later, rats were put back in the conditioning chamber (day 2; No Ext, no tone extinction). The next day, rats were infused with SAL or M&B into LHb (gray arrow) before being presented with tones only (day 3, test), which represents the retrieval of the threat-related memory (red). Right, Coronal drawings of rat brain atlas showing placements of the injector tips in LHb. ***A_2_***, LHb inactivation before retrieval test (day 3) without extinction (gray arrow and shaded area in graph), did not affect defensive responses to the tone, as indicated by similar levels of tone-elicited percent freezing and lever-press suppression ratio (inset, late conditioning; last two-tone trial block and test) between control and experimental groups (SAL, *n* = 8; M&B, *n* = 6). Data are shown as ±SEM in blocks of two trials. Supp. ratio, suppression ratio; x, baseline (pretone) freezing levels. ***B_1_***, left, Schematic representation of open field task. Right, Coronal drawings of rat brain atlas showing the placements of the injector tips in LHb. ***B_2_***, LHb inactivation did not affect locomotion or anxiety-related behavior, as indicated by similar distance traveled and number of entries to the center of the field (SAL, *n* = 12; M&B, *n* = 11). ***C_1_***, left, Schematic representation of lever pressing. Right, Coronal drawings of rat brain atlas showing the placements of the injector tips in LHb. ***C_2_***, LHb inactivation did not affect reward-seeking behavior, as indicated by similar presses per minute to obtain food (SAL, *n* = 5; M&B, *n* = 7). LHb, lateral habenula. Inf, infusion.

To evaluate the necessity of LHb to memory retrieval without tone-associated extinction, we inactivated LHb before memory tests in rats that were not exposed to the tone during extinction (No-Ext; Fig. 3*A1*). Similar to the experiment above, on day 1, rats were threat conditioned to a tone. The next day (day 2), rats were put back in the conditioned chamber without tone exposure. Finally, on day 3, rats were infused with either SAL or M&B into the LHb and tested for memory retrieval to the tone. We found that LHb inactivation before memory test without tone extinction, did not affect retrieval of conditioned tone threat memory (Fig. 3*A2*), as indicated by similar levels of defensive responses across groups during retrieval test (day 3, freezing: *t*_(12)_ = −0.76, *p *=* *0.45; lever-press suppression ratio: *t*_(12)_ = −1.51, *p *=* *0.15). This result is consistent with the interpretation that LHb inactivation effect during retrieval test are not because of impairment on the expression of defensive responses or threat memory retrieval to the conditioned tone and that such effect is extinction-dependent to the tone and not the mere passage of time.

Next, in a different group of animals, we tested the effect of LHb inactivation on basic locomotion activity, an innate aversively motivated behavior (exploration in an open field) and an appetitively motivated behavior. We tested this by infusing SAL or M&B into the LHb before an open field task and before a lever pressing test. We found that LHb inactivation does not affect locomotion, anxiety-related (Fig. 3*B1*,*B2*) or reward-seeking behaviors (Fig. 3*C1*,*C2*), as indicated by similar levels of total distance traveled (*t*_(21)_ = −0.25, *p *=* *0.80) and entries to the center of the open field (*t*_(21)_ = 0.49, *p *=* *0.62), as well as similar levels of presses per minute to obtain food (*t*_(10)_ = 0.04, *p *=* *0.96), respectively. These control experiments suggest that LHb inactivation (on its own) does not affect general movement, hunger, motivation to explore related to anxiety-like behavior or reward-seeking behavior.

### LHb inactivation impairs defensive responses during latent inhibition retrieval

**Figure 4.**
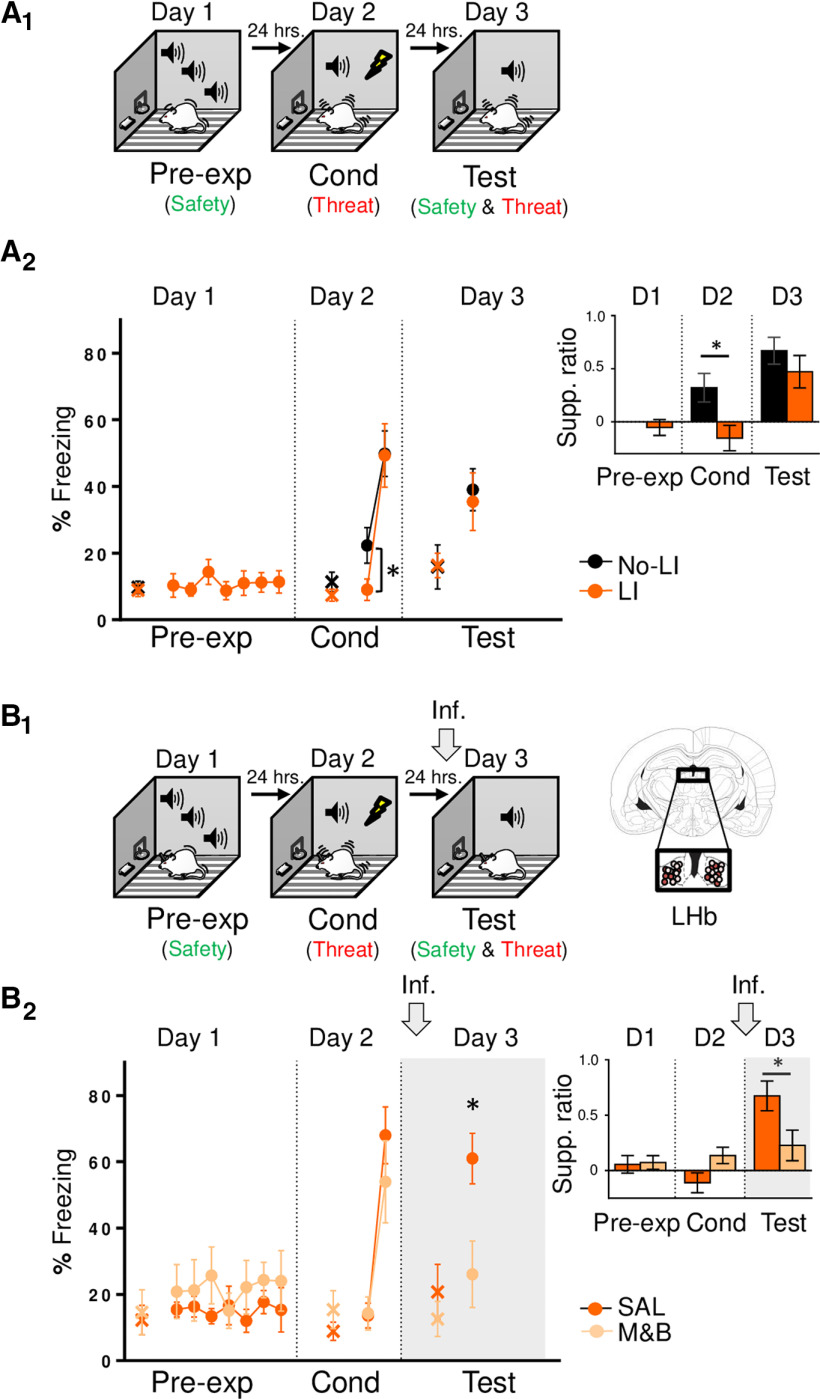
LHb inactivation before retrieval test, after conditioned latent inhibition, abolishes defensive responses. ***A_1_***, Schematic representation of rats training. Rats were either presented with tones (latent inhibition group: LI), which represents safety learning (green), or simply placed in the conditioning chamber (no-latent inhibition group: No-LI; day 1; Pre-exp, preexposure). One day later, both groups were placed in the same behavioral chamber and trained to associate a tone with foot-shocks (day 2; Cond, conditioning), which represents threat learning (red). Finally, the next day, were presented with tones only (day 3, test), which involves the retrieval competition of coexisting safety (green) and threat (red) memories to control defensive responses. ***A_2_***, Tone preexposure weakened subsequent threat learning, as indicated by lower tone-elicited percent freezing and lever-press suppression ratio (inset; late preexposure, early conditioning, and test) levels of the LI group, as compared with No-LI group at the beginning of conditioning (No-LI, *n* = 11; LI, *n* = 12). ***B_1_***, left, Schematic representation of rats training. Rats were pre exposed to tones alone (day 1; Pre-exp, preexposure), which represents safety learning (green). One day later, rats were trained to associate the tone with foot-shocks (day 2; Cond, conditioning), which represents threat learning (red). Finally, the next day, rats were infused with SAL or M&B into LHb (gray arrow) before being presented with tones only (day 3, test), which involves the retrieval competition of coexisting safety (green) and threat (red) memories to control defensive responses. Right, Coronal drawings of rat brain atlas showing placements of the injector tips in LHb. ***B_2_***, LHb inactivation before retrieval test (day 3), after latent inhibition and conditioning (gray arrow and shaded area in graph), decreased defensive responses as indicated by lower levels of tone-elicited percent freezing and lever-press suppression ratio [inset; late conditioning (last two-tone trial block), early extinction (first block of trails), and test] in the experimental group as compared with the control group (SAL, *n* = 8; M&B, *n* = 5). Data are shown as ±SEM in blocks of two trials. LHb, lateral habenula. Inf, infusion. Supp. ratio, suppression ratio; x, baseline (pretone) freezing levels; *p < 0.05.

Together, our above results are consistent with the idea that rather than being necessary for simple task performance (freezing or not to the tone), LHb-mediated activity is crucial for flexible and adaptive behaviors during more complex situations in mammals (Baker et al., 2017). Threat and extinction learning and memory as well as expression of defensive responses by themselves may represent relatively simple situations as compared with flexibly selecting the adaptive emotional memory (threat or safety) to control (promote or suppress) the expression of defensive behaviors. Such a situation occurs when coexisting threat and safety memories compete for control of behavior after extinction training. To further test the idea that LHb is necessary in conditions where coexisting threat and safety memories compete for control of behavior, we switched the order of opposing emotional learning experiences (safety learning followed by threat learning rather than threat learning followed by safety learning) by using latent inhibition (Fig. 4*A1*). To do this, on day 1, rats were either trained to acquire a latent inhibition-mediated safety memory by presenting tones without foot-shocks (latent inhibition group: LI) or simply placed in the conditioning chamber (no tones or foot-shocks; no latent inhibition group: No-LI). On day 2, both group of rats were threat conditioned to the tone. One day later (day 3), rats were tested for memory retrieval to the tone. As expected, we found that preexposure to a tone, weakened subsequent threat conditioning to tones (Fig. 4*A2*), as indicated by lower defensive response levels of the LI group with respect to No-LI group at the beginning of conditioning (first two-tone trial block; freezing: No-LI: 22.30%; LI: 9.03%; *t*_(21)_ = −2.16, *p *=* *0.04; lever-press suppression ratio: No-LI: 0.32; LI: −0.15; *t*_(21)_ = −2.64, *p *=* *0.01). On day 3, both groups showed similar defensive response levels (freezing: *t*_(21)_ = −0.33, *p *=* *0.74; lever-press suppression ratio: *t*_(21)_ = −0.98, *p *=* *0.33). This result suggests that cued-mediated latent inhibition leads to the formation of a safety memory that retards conditioning learning to the tone. Next, to evaluate the necessity of LHb during the competition of coexisting latent inhibition-mediated safety memory and conditioned-mediated threat memory, we pharmacologically inactivated the LHb before a postconditioning memory test (Fig. 4*B1*). On day 1, rats were preexposed to the tone. The next day (day 2), rats were conditioned to the tone by paring tones with foot-shocks. Finally, on day 3, rats were infused with either SAL or M&B into the LHb and tested for memory retrieval to the tone. Similar to infusion during retrieval after extinction, we found that LHb inactivation before memory test, after conditioning, decreased defensive response levels of the experimental group as compared with control group during memory test (day 3, freezing: SAL: 61.00%, M&B: 26.09%; *t*_(11)_ = −2.80, *p *=* *0.01; lever-press suppression ratio: SAL: 0.65, M&B: 0.22; *t*_(11)_ = −2.25, *p *=* *0.04; Fig. 4*B2*). Since we ruled out above that LHb inactivation impairs the expression of defensive responses and threat memory retrieval, this finding supports the idea that LHb inactivation before the retrieval test biased toward facilitated the expression of the latent inhibition-mediated safety memory over the conditioned-mediated threat memory. This last interpretation suggests that, independently of when those opposing emotional memories are formed (safety before threat memory or threat before safety memory), LHb is necessary to regulate competing and coexisting threat and safety memories by promoting defensive responses to ultimately exert control over adaptive behaviors.

### LHb inactivation switches choice behavior bias from avoidance to reward-seeking

**Figure 5.**
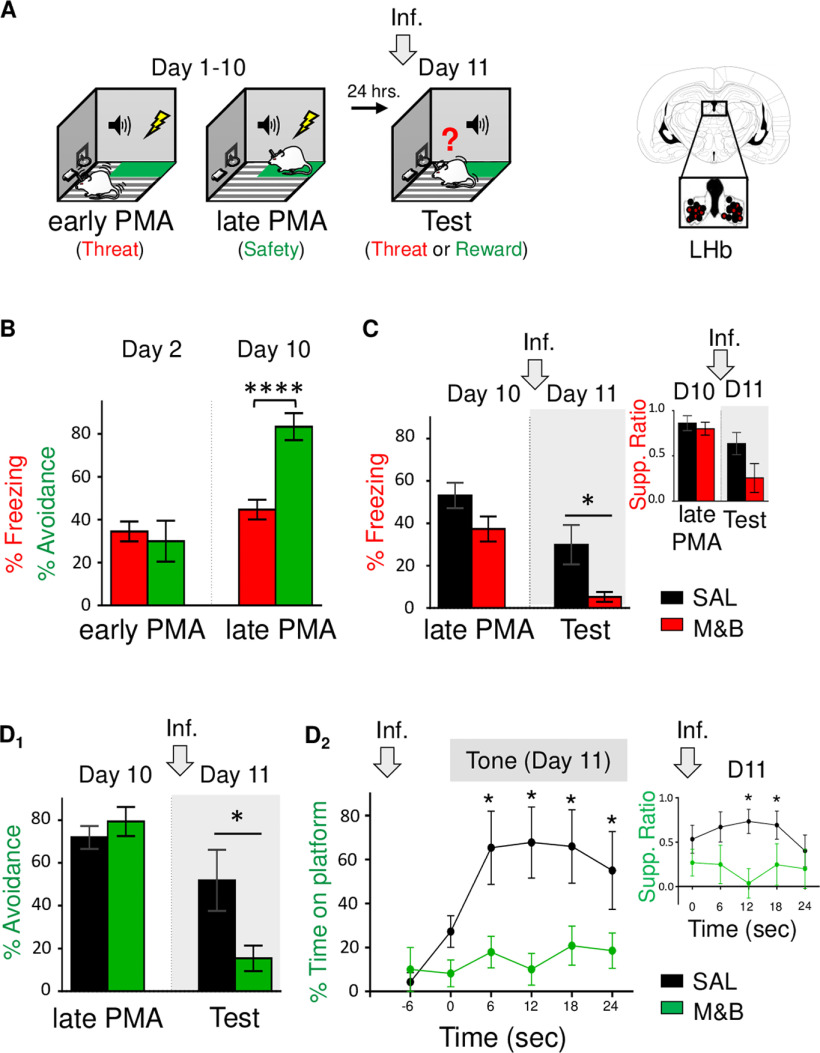
LHb inactivation before PMA retrieval test biases choice-mediated defensive behavior toward reward-seeking. ***A***, left, Schematic representation of rats training. Rats were trained to associate tone with foot-shocks as well as associating the tone with moving to a safety platform learn to avoid the shock (day 1–10; PMA). Early PMA (day 2) represents classical conditioned-mediated threat learning (red), whereas late PMA (day 10) represents instrumental avoidance-mediated safety learning (green). One day later, rats were infused with SAL or M&B into LHb (gray arrow) before being presented with tones only (day 11, test), which involves the retrieval competition of coexisting threat (red) and safety (green) memories to control choice behavior. Right, Coronal drawings of rat brain atlas showing placements of the injector tips in LHb. ***B*,** During early PMA training (day 2), freezing and avoidance levels are similar. In contrast, during late PMA training (day 10), avoidance levels are higher than freezing levels. Thus, avoidance levels dramatically increased with PMA training while freezing levels remain similar (*n* = 15). ***C***, LHb inactivation before PMA test (gray arrow and shaded area in graph), impaired retrieval of PMA memory, as indicated by lower tone-elicited percent freezing responses (but not suppression ratio) of the experimental group as compared with control group (SAL, *n* = 7; M&B, *n* = 8). ***D*_,_** LHb inactivation before PMA test (gray arrow) biased choice-mediated avoidance toward reward-seeking behavior as indicated by lower tone-elicited percent avoidance (***D_1_***) and time spent in platform (***D_2_***), as well as lower suppression ratio (inset) in the experimental group as compared with the control group (SAL, *n* = 7; M&B, *n* = 8). Data are shown as ±SEM in blocks of two trials in ***B***, ***C***, ***D_1_*** and in 6-s bins in ***D_2_***. LHb, lateral habenula. Inf, infusion. Supp. ratio, suppression ratio; ****p < 0.00001, *p < 0.05.

Besides influencing adaptive defensive responses triggered by threats like freezing (this study and Martínez-Canabal et al., 2019) and avoidance (Thornton and Bradbury, 1989; Tomaiuolo et al., 2014; Trusel et al., 2019), LHb activity has been recently implicated in biasing choice behavior (Stopper and Floresco, 2014; Baker et al., 2017; Mathis et al., 2017). Therefore, using PMA task (Bravo-Rivera et al., 2014; Diehl et al., 2019; Bravo-Rivera and Sotres-Bayon, 2020), we tested the effect of LHb inactivation when rats are challenged to choose between taking action to avoid a threat or taking action to approach food guided by competing coexisting threat and safety memories. After being trained to press a lever to obtain food, rats were trained for 10 d to learn to avoid a threat (foot-shock) signaled by a tone, by stepping into the safe platform (Fig. 5*A*). During early PMA training (day 2), rats acquired classical conditioning (tone-shock threat association), but barely any instrumental learning (tone-platform safety association), as indicated by moderate levels of freezing and relatively low levels of avoidance responses (*t*_(28)_ = 0.42, *p *=* *0.67; Fig. 5*B*), respectively. In contrast, by late PMA (day 10), freezing responses remained similar to early PMA (34.49% on day 2 vs 44.71% on day 10; *t*_(28)_ = −1.56, *p *=* *0.12), but avoidance responses dramatically increased (30.00% on day 2 vs 83.33% on day 10; *t*_(28)_ = −4.67, *p *<* *0.00006), as indicated by moderate levels of freezing and high levels of avoidance responses (freezing: 44.71% and avoidance: 83.333%; *t*_(28)_ = −4.95, *p *<* *0.000006). These results suggest the formation of a tone threat memory occurs during early PMA, whereas the establishment of a tone safety memory during late PMA. Finally, the next day (day 11), rats were infused with either SAL or M&B into the LHb and tested for choice behavior (avoid or approach to obtain food) guided by competing threat and safety memories. Notably, we found that LHb inactivation before PMA retrieval test, impaired defensive responses, as indicated by decreased freezing and avoidance responses of the experimental group as compared with control group during PMA test [freezing: SAL: 29.85%, M&B: 5.22%; *t*_(13)_ = −2.73, *p *=* *0.01 (Fig. 5*C*); avoidance SAL: 59.26%, M&B: 15.39%; *t*_(13)_ = −3.00, *p *=* *0.01 (Fig. 5*D1*)]. Although showing a similar decrease trend, averaged lever press suppression ratio during test was not significantly different after inactivation (SAL: 0.63, M&B: 0.25; *t*_(13)_ = 1.85, *p *=* *0.08; Fig. 5*C*, inset). However, analyzing the percent time spent on the platform during the tone and the suppression ratio in 6-s bins, revealed that LHb inactivation blocked both avoidance and suppression ratios (i.e., inactivation facilitated lever press suppressing during tone compared with pretone) compared with animals in the control group [time in platform: *F*_(1,13)_ = 7.50, *p *=* *0.01; *post hoc* comparisons: −6 to −1 s, *p *=* *0.71; 0–6 s, *p *=* *0.23; 7–12 s, *p *=* *0.0048; 13–18 s, *p *=* *0.0008; 19–24 s, *p *=* *0.007; 25–30 s, *p *=* *0.02 (Fig. 5*D2*); suppression ratio: *F*_(1,13)_ = 6.55, *p *=* *0.02; *post hoc* comparisons: 0–6 s, *p *=* *0.32; 7–12 s, *p *=* *0.11; 13–18 s, *p *=* *0.0042, 19–24 s, *p *=* *0.03; 25–30 s, *p *=* *0.44 (Fig. 5*D*, inset)]. In sum, LHb inactivation biased the ability to actively choose between avoiding a threat or approaching food, toward driving reward-seeking behavior, suggesting that LHb is necessary to bias choice toward the promotion of defensive responses when competing threat and safety memories guide motivated behavior.

## Discussion

We investigated the conditions under which LHb is necessary to regulate defensive responses to a threat. By combining pharmacological inactivations with various behavioral paradigms in rats, including tasks that involve simultaneous retrieval of competing threat and safety memories, our study reveals a previously unknown role for the LHb in defensively responding to a cue that predicts outcomes with opposing emotional valences (threat and safety) during conflict. These findings add to a growing body of evidence showing that LHb is involved in the regulation of aversively motivated behaviors and highlight emotional conflict as a condition under which this brain region is critical to promote defensive responses.

### LHb promotes defensive strategies during threat/safety memory conflict

Even after successful extinction learning to threat, conditioned defensive responses often return with the simple passage of time (Rescorla, 2004; Sotres-Bayon et al., 2006), even after a single day (Quirk, 2002). By using c-Fos as neural activity marker, a previous study identified that high LHb activity is positively correlated with high threat recovery after extinction (Martínez-Canabal et al., 2019). Consistently, in this study, we found that LHb inactivation prevents the recovery of extinguished defensive responses. Together, these findings indicate that LHb is not only recruited but is necessary to prevent the expression of the extinction memory thereby allowing defensive responses to return after extinction.

LHb activity may normally prevent the retrieval of safety memories in the service of driving defensive responses by suppressing downstream structures involved in appetitive motivated behaviors. A recent study found that optogenetic stimulation of dopaminergic activity in the ventral tegmental area (VTA), a prominent LHb projection site, facilitates extinction memory (Salinas-Hernández et al., 2018). Another recent study showed that chemogenetic inhibition of serotonergic activity of dorsal raphe receiving LHb input provides animals with resilience during aversive conditions (Varga et al., 2003; Andalman et al., 2019). Because LHb is known to potently inhibit brainstem dopaminergic and serotoninergic activity (Varga et al., 2003; Matsumoto and Hikosaka, 2007; Lammel et al., 2012; Andalman et al., 2019), it is possible that suppressing LHb activity disinhibits these monoaminergic pathways thereby facilitating retrieval of safety memories (extinction and latent inhibition) and resilience in aversive conditions. In line with these findings, we suggest that LHb activity normally prevents expression of the extinction memory possibly by inhibiting VTA dopaminergic activity in the service of signaling the prevailing threat memory during recovery. This interpretation is further consistent with recent findings that indicate that LHb activity increases under aversive conditions, leading individuals toward negative expectations (Shabel et al., 2019). Furthermore, blockade of LHb activity with ketamine results in antidepressant effects (Yang et al., 2018; Cui et al., 2019) and decreased LHb activity mediated by enhanced neurogenesis prevents threat recovery (Martínez-Canabal et al., 2019). Thus, we suggest that when LHb is suppressed (with pharmacological inactivation, ketamine or enhanced neurogenesis), the negative expectation signal (worse than expected or “pessimism”) is blocked (likely by disinhibiting monoaminergic pathways involved in motivation), thereby allowing individuals to be able to explore other options such as approaching food when available.

Extinction allows to evaluate how coexisting threat and extinction memories compete for the control of defensive responses (Rescorla, 2001; Myers and Davis, 2002; Quirk, 2002; Quirk and Mueller, 2008; Herry et al., 2010; Martínez-Canabal et al., 2019), and our findings suggest that the LHb is a critical brain region necessary to promote defensive responses during retrieval of conflicting threat and extinction memory. This interpretation is consistent with the idea that LHb activity is shaped by previous aversive and appetitive emotional associations (Wang et al., 2017) and that experience-dependent modifications in this brain region are necessary for the selection of defensive responses (Amo et al., 2014). Together, these findings suggest the possibility that LHb is involved in promoting defensive responses in conditions where memory retrieval is guided by coexisting previous aversive and appetite experiences. Another condition that allows us to evaluate the competition of threat and safety memories, although in the inverse order, is following latent inhibition. We found that independently of the order of acquisition of threat and safety learning, LHb is necessary to guide defensive responses mediated by the competition of coexisting emotional experiences (threat vs safety or safety vs threat). Thus, together these results indicate that, LHb may not mediate recovery of extinguished defensive responses per se but rather the promotion of threat-related behaviors in retrieval events involving competition of threat and safety memories. Further support for this conclusion, could include experiments that involve testing cued memory retrieval after extinguishing in a different context, as well as assessing threat/safety conflict retrieval using two separately trained cues (e.g., tone for threat and light for safety), rather than one (same tone for threat and safety).

In future studies, it will be important to characterize how information about threat and safety memories are integrated at the LHb to regulate defensive responses during conflict. LHb receives strong projections from regions of the medial prefrontal cortex that are involved in dual control of defensive responses (Kim and Lee, 2012). The prelimbic prefrontal region promotes conditioned defensive responses, whereas the infralimbic prefrontal region suppresses them (for review, see Sotres-Bayon and Quirk, 2010). Further, recent studies showed that prefrontal connectivity with LHb is crucial for flexibly responding to learned information (Mathis et al., 2017; Mathis and Lecourtier, 2017). This raises the possibility that a prefrontal-habenular circuit may flexibly control defensive responses when threat and safety memories are in conflict.

### LHB promotes defensive responses during conflict-mediated choice behavior

Our finding that LHb is necessary for retrieval of PMA is consistent with previous studies that show that LHb is necessary for avoidance learning (Wilcox et al., 1986; Thornton and Bradbury, 1989). However, PMA differs from traditional avoidance tasks in that it uses choice behavior to evaluate competing emotional memories during conflict (Bravo-Rivera et al., 2014; Diehl et al., 2019; Bravo-Rivera and Sotres-Bayon, 2020). During PMA training, a platform acquires a positive motivational value (safety) while the grid acquires a negative value (threat). Then rats are challenged, during PMA retrieval (no shock delivered), to decide, guided by competing emotional memories signaled by a tone, whether to step onto the safe platform or press a lever to obtain food. Normally, rats choose to avoid the shock by stepping into the platform, however LHb inactivated rats pressed the lever to obtain food despite the threat. This finding suggests that LHb is necessary to promote defensive responses over reward-seeking behavior during conflict choice guided by memories with opposing motivational valences (threat and safety memories).

A previous study reported increase activity of dopaminergic neurons that allow successful avoidance responses (Oleson et al., 2012). The LHb inhibits dopaminergic VTA activity, through GABAergic neurons in the tail of this brain structure (the rostromedial tegmental nucleus), promoting defensive responses, thereby putting a “break” on reward-seeking (Jhou et al., 2009; Quirk and Sotres-Bayon, 2009; Proulx et al., 2018). Our pharmacological inhibition of the LHb may allow disinhibition of VTA dopaminergic activity leading to a facilitation of reward-seeking thereby making the individual more prone to risky behaviors. This is consistent with a previous study showing that LHb inactivation increased hesitation to make a choice between risky reward options associated with different subjective values (Stopper and Floresco, 2014) and the notion that LHb is involved in guiding survival decisions based on stimuli with learned motivation value (Hikosaka, 2010; Baker et al., 2016). Thus, our PMA findings support the notion that LHb is critical for biasing choice behavior mediated by value-based decision-making and highlight its fundamental role in promoting defensive behavioral strategies during conflict-mediated choice behavior.

### LHb as a critical node in emotional conflict

Overall, our study reveals that emotional memory conflict is a fundamental survival condition under which LHb is required to promote defensive strategies. This conclusion is consistent with the emerging notion that LHb acts as a critical node in the control of emotional behaviors guided by aversive events in health and disease, at least in part, mediated by regulating downstream midbrain dopaminergic and serotoninergic structures (Hu et al., 2020). We propose that during emotional conflict, LHb activity is required to bias experience-dependent emotional behavior toward defensive behavioral responses and away from reward-seeking behavior, likely by suppressing downstream structures involved in appetitive motivated behaviors. Accordingly, a lack of activity in LHb during emotional conflict results in a shift toward reward-seeking behavior and away from defensive responding, likely through disinhibition of downstream structures involved in appetitive motivated behaviors. Identifying the brain regions embedded in a complex network that are required for conflict-mediated behavioral responses, may be key for understanding how the brain balances information about competing emotional memories to adaptatively regulate emotional behaviors in health and disease.

## Synthesis

Reviewing Editor: Quentin Pittman, University of Calgary Decisions are customarily a result of the Reviewing Editor and the peer reviewers coming together and discussing their recommendations until a consensus is reached. When revisions are invited, a fact-based synthesis statement explaining their decision and outlining what is needed to prepare a revision will be listed below. The following reviewer(s) agreed to reveal their identity: Sheri Mizumori. Note: If this manuscript was transferred from JNeurosci and a decision was made to accept the manuscript without peer review, a brief statement to this effect will instead be what is listed below.

While it has been previously shown that the LHb drives fear in most, but not all, paradigms, exploring its role in fear inhibition memories has not been systematically tested, as it is here. While it is generally thought that the lateral habenula serves as an aversion signal, its contribution to behaviors such as threat is not clear. Here it is shown that the lateral habenula becomes involved only in certain threatening (i.e. aversive) situations, and that is when there are competing memories (e.g. threat vs safety).

Both reviewers agree that your conclusions are solid and your paper makes a useful contribution to the field without the need for additional experiments. Thus, it is potentially acceptable pending attention to a number of the critical suggestions for improvement that involve elaboration on some of the themes in the intro/discussion, increasing the clarity of the graphs and visual abstract, correcting the typographical errors and providing missing information. The two reviews that follow clarify these deficiencies:

Reviewer 1.

The lateral habenula (LHb) has long been implicated in negative signaling and guiding behavioral responses following aversive events. However, the role of the LHb in the regulation of defensive responses is not clear. Given that the LHb also has important roles in positive signaling, the authors aimed to characterize the LHb’s role in influencing defensive responses during competing threat and safety memories. An elegant series of experiments combined pharmacological inactivation with strategic behavioral paradigms that involved threatening stimuli and appetitive motivation. The first experiment showed that LHb inactivation prior to auditory threat conditioning did not impair threat learning and memory. The second experiment showed that LHb inactivation prior to extinction training following threat conditioning did not impair threat memory retrieval or extinction learning and memory. A third experiment showed that LHb inactivation prior to retrieval memory and following extinction training facilitated extinction memory, more so than controls. The fourth experiment showed that inactivating the LHb prior to the memory test in rats that were not exposed to the tone during extinction did not affect retrieval of conditioned tone threat memory. In the fifth experiment, LHb inactivation prior to an open field task and lever pressing test did not affect locomotion or motivation. The sixth experiment used a latent inhibition paradigm to assess whether or not the LHb is necessary in conditions where threat and safety memories are in competition. Inactivating the LHb prior to the post-conditioning memory test revealed a decrease in defensive response levels. Lastly, the seventh experiment used a platform-mediated avoidance (PMA) paradigm to test choice behavior in the face of competing threat and safety memories. LHb inactivation prior to the PMA retrieval test resulted in impaired defensive responses. The authors concluded that the LHb mediates defensive responses but only when threat and safety memories compete to bias choice outcomes.

General comments: The traditional view that the LHb primarily serves to signal aversive cues is inadequate. In this study, the results of the specific combination of behavioral assessments generated compelling evidence that there are specific task conditions that require the LHb to guide memory-driven behaviors: critical for LHb engagement is the presence of competing threat and safety memories. This novel insight can help to explain the otherwise seemingly disparate LHb behavioral results, and it can be used to guide future LHb analyses, especially those relevant to clinical conditions such as depression.

Specific comments:

1. To provide the reader with a more complete model of LHb function, it is suggested that the authors provide a short description of how task-relevant information arrives in the LHb in order to influence down-stream monoamine systems for behavior (Mathis et al., 2017).

2. A total of 144 rats were used for the entire study, but it was not clear how many rats were used (reused?) for each experiment.

3. For clarity in figure 1a2, add a legend for the X, denoting it as baseline.

4. In figure 1b2, the M&B baseline freezing percentage is 10% greater than the control group. There was no explanation for this phenomenon in the text.

5. It would be helpful to provide a supplementary histological image to illustrate the localization of lateral habenula inactivation with M&B.

6. The miss M&B group shows that hippocampal inactivation had no effects. Is there evidence that the medial habenula similarly plays no role? The M&B injections could have diffused to the neighboring medial habenula.

7. Line 418: what does ’emotional conflict’ refer to in the context of this study?

There are many typos. For example:

8. Line 100: Is the D/V coordinate from top of brain or skull?

9. Line 145: change ’allowed’ to allow

10. Line 187: change ’absent’ to absence

11. Line 198: change ’the percent of rat’s to the percentage of rats

12. Line 199: change ’into’ to on

13. Line 295: change ’test’ to tests

14. Line 304: change ’effect during retrieval test is’ to effects during retrieval tests are

15. Line 313: change ’LHb does’ to LHb inactivation does

16. Line 318: change ’anxiety like-’ to anxiety-like

17. Line 360: change ’test biased toward facilitating’ to test facilitated

18. Line 376: change ’After trained’ to After being trained

19. Line 378: change ’acquire’ to acquired

20. Line 387: change ’memory during’ to memory occurs during

21. Line 398: change ’spent in’ to spent on the

22. Line 399: change ’suppression’ to the suppression

23. Line 400: change ’ratio’ to ratios

24. Lines 461-462, 466-468, 473-474, 476-477: within a single paragraph, the statement “the LHb is necessary to guide behaviors during competition of existing fear/safety memories” is restated four times.

25. Line 703: change “grayed” to shaded

Reviewer 2:

This study used pharmacological inactivation to assess the role of the LHb in 3 different fear regulation tasks: extinction, latent inhibition, and platform-mediated avoidance. The authors systematically inactivated the LHb across the learning and memory timeline, with inactivations happening either before fear training, extinction training or memory retrieval. All of the effects were during memory retrieval sessions when the LHb was inactivated. Collectively, the data are compelling in showing that LHb inactivation results in downregulated fear, indicating the LHb drives cued fear responses when undisturbed.

Most interesting was that these effects were consistent across multiple types of fear regulation tasks. This suggests that the LHb must be a critical node in the network controlling fear output. The discussion could be strengthened by elaborating more on how the LHb is achieving this (there was some discussion on disinhibition of VTA, but I think the authors could do more).

The usage of ’safety’ to describe some of their tasks is confusing at times. For example, in the abstract text the authors do not call extinction ’safety’ but in the graphical abstract it is focused on ’safety’. Moreover, in the graphical abstract it looks like distinct, separate cues are serving as threat and safety (i.e. CS+ vs CS-) cues. Further, the authors oftentimes refer to the memory test as having the fear and safety memories in conflict or competing. Even though extinction is widely considered to produce a separate memory for a CS/no threat association, describing that memories for the threat conditioning and extinction conditioning are in conflict may be misleading because there is no direct way to show the 2 memories are in fact active and in conflict. A simpler approach to assessing threat/safety conflict would have been to train 2 separate cues (e.g. tone for threat, light for safety) and then present them in compound, in direct conflict, during the test session. Based on the results presented the prediction would be LHb inactivation would show significantly less fear to a threat/safety cue versus saline. Another way to have created more direct conflict would have been to extinguish in a different context than threat conditioning, and then completed the memory retrieval with the extinguished cue in the threat context. This reliably induces rebound of fear in most experiments; if the authors could have blocked this rebound with LHb inactivation, this would have been stronger support for their conclusions. These considerations should be discussed in the discussion.

LINE 259: “...abolished defensive responses (freezing and pressing)....” Should this be ’suppressed pressing?

LINE 328-329: grammatical typo “Such situation occurs....”

## Author Response

POINT BY POINT REPLY TO REVIEWERS

Comments on the Visual Abstract for Author (Required):

Needs attention as indicated in the review

As suggested by Reviewer #2, we modified the Visual abstract to denote that the

same sensory auditory stimulus (tone) served as both threat and safety cue.

Reviewer 1.

Specific comments:

1. To provide the reader with a more complete model of LHb function, it is suggested

that the authors provide a short description of how task-relevant information arrives

in the LHb in order to influence down-stream monoamine systems for behavior

(Mathis et al., 2017).

As suggested by the Reviewer, we added a short discussion of how task-relevant

input to LHb may influence defensive behaviors (lines 482-491).

2. A total of 144 rats were used for the entire study, but it was not clear how many

rats were used (reused?) for each experiment.

We used different groups of rats for each experiment (total of 144 rats: Fig. 1A, n =

12; Fig. 1B, n = 20; Fig. 2A, n = 24; Fig. 3A, n = 14; Fig. 3B, n = 23; Fig. 4A, n = 23;

Fig. 4B, n = 13; Fig. 5, n = 15), except for the lever pressing test (Fig. 3C). For this

test, we analyzed presses per minute before conditioning (pretone lever pressing)

taken from rats in the first experiment (Fig. 1A). We now include this information in

the Methods. We thank the Reviewer for noticing the missing information.

Additionally, we replaced Figure 3C1 after noticing misplacements in the brain

illustration.

3. For clarity in figure 1a2, add a legend for the X, denoting it as baseline.

To maintain figure simplicity without adding more legends, we opted to describe in

the appropriate figure legends (Figs. 1,2,3 and 4) that “x” in graphs represent

baseline (pretone) freezing levels.

4. In figure 1b2, the M&B baseline freezing percentage is 10% greater than the

control group. There was no explanation for this phenomenon in the text.

The Reviewer is correct that baseline pretone freezing percentages during drug-free

memory test on day 3 (Figure 1B2) appear to be higher in the M&B group as

compared to the SAL group. We re-inspected the individual data points from Figure

1B2 (see Author response image 1 below, with individual data points from Figure

1B2) and found that the mentioned trend appears to be explained by a rat in the

experimental group (circled in red) that showed unusually high freezing (97%)

compared to the rest of the animals in the experiment. The apparent trend, however,

is not significantly different (t(18) = 1.36, p = 0.18). Consistently, we found that lever

pressing at this point was not significantly different between groups either (t(18) = -

0.28, p = 0.78). These results indicate that LHb inactivation before extinction learning

did not affect retrieval of contextual extinction memory. For clarity, we modified the

Methods noticing that in all experiments pretone freezing percentages were not

significantly different between experimental and control groups (all p values > 0.05).

2

5. It would be helpful to provide a supplementary histological image to illustrate the

localization of lateral habenula inactivation with M&B.

At eNeuro submitting supplementary material is not an option. As suggested by the

Reviewer, below we provide a histological image to illustrate localization of lateral

habenula infusions (Author response image 2). The figure below shows a

representative rat brain coronal section with cannula placement localized in the

lateral habenula (LHb). Left, Representative photomicrograph of Nissl-stained

coronal section (45 μm) from a brain of a rat with cannula placement localized in the

LHb. Right, Rat brain atlas coronal section (-3.6 mm relative to bregma; adapted

from Paxinos and Watson, 1998) illustrating localization and boundaries of the LHb.

6. The miss M&B group shows that hippocampal inactivation had no effects. Is there

evidence that the medial habenula similarly plays no role? The M&B injections could

have diffused to the neighboring medial habenula.

Because we did not have rats with bilateral medial habenula (MHb) misplacements

in the miss M&B group we cannot exclude (using this experimental strategy) that

inactivations did not diffuse to MHb. However, using a different strategy (evaluating

c-Fos expression in a subset of rats from this experiment (one SAL and one M&Btreated rat)), we found that after SAL infusion, the LHb is heavily recruited during

extinction memory retrieval, whereas MHb is not (see Author response image 3

below, with behavioral data from Fig 2A2). This result is consistent with a previous

study showing recruitment of LHb during recovery of an extinguished threat memory

(Martinez-Canabal et al., 2019), and suggests that MHb does not play a role in

extinction memory retrieval. Accordingly, we found that inactivation silenced

recruitment of LHb, while c-Fos expression in MHb did not change. Thus, even

though we cannot directly rule out the spread of infusions to MHb, results from our

3

pilot experiment suggest that inactivations specifically silenced extinction retrievalmediated activity of LHb. This also further supports the conclusion that the dosage

and volume used in this report and a previous study (Stopper and Floresco 2014)

effectively inactivated LHb. We now include this information in the Methods.

7. Line 418: what does ’emotional conflict’ refer to in the context of this study?

We modified this line to clarify what we refer to as “emotional conflict” in the context

of our study.

There are many typos. For example:

8. Line 100: Is the D/V coordinate from top of brain or skull?

D/V coordinates were taken from the skull. We modified the text in the manuscript

accordingly.

9. Line 145: change ’allowed’ to allow

10. Line 187: change ’absent’ to absence

11. Line 198: change ’the percent of rat’s to the percentage of rats

12. Line 199: change ’into’ to on

13. Line 295: change ’test’ to tests

14. Line 304: change ’effect during retrieval test is’ to effects during retrieval tests are

15. Line 313: change ’LHb does’ to LHb inactivation does

16. Line 318: change ’anxiety like-’ to anxiety-like

17. Line 360: change ’test biased toward facilitating’ to test facilitated

18. Line 376: change ’After trained’ to After being trained

19. Line 378: change ’acquire’ to acquired

20. Line 387: change ’memory during’ to memory occurs during

21. Line 398: change ’spent in’ to spent on the

22. Line 399: change ’suppression’ to the suppression

23. Line 400: change ’ratio’ to ratios

We corrected these typos. We thank the Reviewer for careful revision of the

manuscript text.

4

24. Lines 461-462, 466-468, 473-474, 476-477: within a single paragraph, the

statement “the LHb is necessary to guide behaviors during competition of existing

fear/safety memories” is restated four times.

The Reviewer is correct in that repeating this statement in the same paragraph was

unnecessary. We modified the Discussion accordingly.

25. Line 703: change “grayed” to shaded

As suggested by the Reviewer, we made this change in all figure legends.

Reviewer 2:

Most interesting was that these effects were consistent across multiple types of fear

regulation tasks. This suggests that the LHb must be a critical node in the network

controlling fear output. The discussion could be strengthened by elaborating more on

how the LHb is achieving this (there was some discussion on disinhibition of VTA,

but I think the authors could do more).

As suggested by the Reviewer, we extended the discussion on how LHb may

mediate defensive behaviors (lines 482-491).

The usage of ’safety’ to describe some of their tasks is confusing at times. For

example, in the abstract text the authors do not call extinction ’safety’ but in the

graphical abstract it is focused on ’safety’.

We modified the Abstract to clarify the usage of the term “safety” by referring to

threat conditioning as fear and threat extinction as safety, and in the next sentence

referring to both extinction and latent inhibition as safety memories.

Moreover, in the graphical abstract it looks like distinct, separate cues are serving as

threat and safety (i.e. CS+ vs CS-) cues.

We modified the Graphical Abstract to denote that the same sensory auditory

stimulus (tone) served as threat and safety cue.

Further, the authors oftentimes refer to the memory test as having the fear and

safety memories in conflict or competing. Even though extinction is widely

considered to produce a separate memory for a CS/no threat association, describing

that memories for the threat conditioning and extinction conditioning are in conflict

may be misleading because there is no direct way to show the 2 memories are in

fact active and in conflict. A simpler approach to assessing threat/safety conflict

would have been to train 2 separate cues (e.g. tone for threat, light for safety) and

then present them in compound, in direct conflict, during the test session. Based on

the results presented the prediction would be LHb inactivation would show

significantly less fear to a threat/safety cue versus saline. Another way to have

created more direct conflict would have been to extinguish in a different context than

threat conditioning, and then completed the memory retrieval with the extinguished

cue in the threat context. This reliably induces rebound of fear in most experiments;

if the authors could have blocked this rebound with LHb inactivation, this would have

been stronger support for their conclusions. These considerations should be

discussed in the discussion.

We agree that the experiments suggested by the Reviewer would provide further

support to our conclusions. We include these considerations in the discussion (lines

477-481).

5

LINE 259: “...abolished defensive responses (freezing and pressing)....” Should this

be ’suppressed pressing?

We corrected omission of the word “suppressed” in this sentence.

LINE 328-329: grammatical typo “Such situation occurs....”

We corrected the grammatical typo in this sentence.
